# Cell-free strategies for cardiomyocyte proliferation and heart repair

**DOI:** 10.1007/s43440-025-00805-7

**Published:** 2025-11-05

**Authors:** Agnieszka Łoboda, Tomasz Zieliński, Józef Dulak

**Affiliations:** Department of Medical Biotechnology, Faculty of Biochemistry, Biophysics and Biotechnology, Jagiellonian University, Kraków, Poland

**Keywords:** Cardiomyocytes, Heart regeneration, Heart failure, Myocardial infarction

## Abstract

Heart regeneration, or the replacement or restoration of damaged myocardium, remains one of the most challenging areas in regenerative medicine, primarily due to the limited regenerative capacity of the adult human heart. Unlike the embryonic heart, which exhibits robust cardiomyocyte proliferation, postnatal cardiac muscle cells permanently exit the cell cycle, resulting in minimal regenerative potential following injury such as myocardial infarction. This limitation contributes significantly to the progression of heart failure, a leading cause of morbidity and mortality worldwide. Recent breakthroughs in understanding the molecular and cellular mechanisms that govern cardiomyocyte proliferation have revealed that targeting signaling pathways (e.g., Hippo-YAP), cell cycle regulators, epigenetic modulators, and extracellular components may be a promising strategy for stimulating heart repair. Despite these advances, cardiac regeneration still faces significant obstacles in replacing damaged tissue and ensuring the regenerated muscle functions effectively within the complex heart system. This review aims to provide a comprehensive analysis of emerging regulatory mechanisms involved in cardiomyocyte proliferation and myocardial regeneration. It critically evaluates current strategies for promoting heart regeneration, with particular emphasis on the most promising molecular pathways and therapeutic approaches with translational potential. Ongoing research, as summarized in this review, continues to expand the potential of regenerative medicine to repair heart damage, offering hope for more effective treatments for heart disease.

## Graphical Abstract

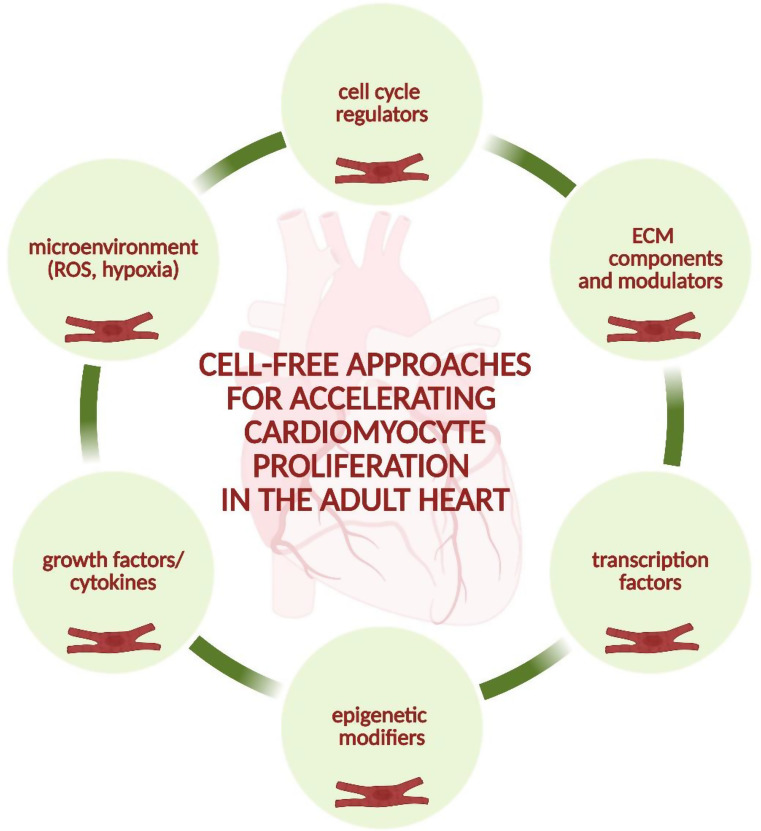

## Introduction

**Fig. 1:**
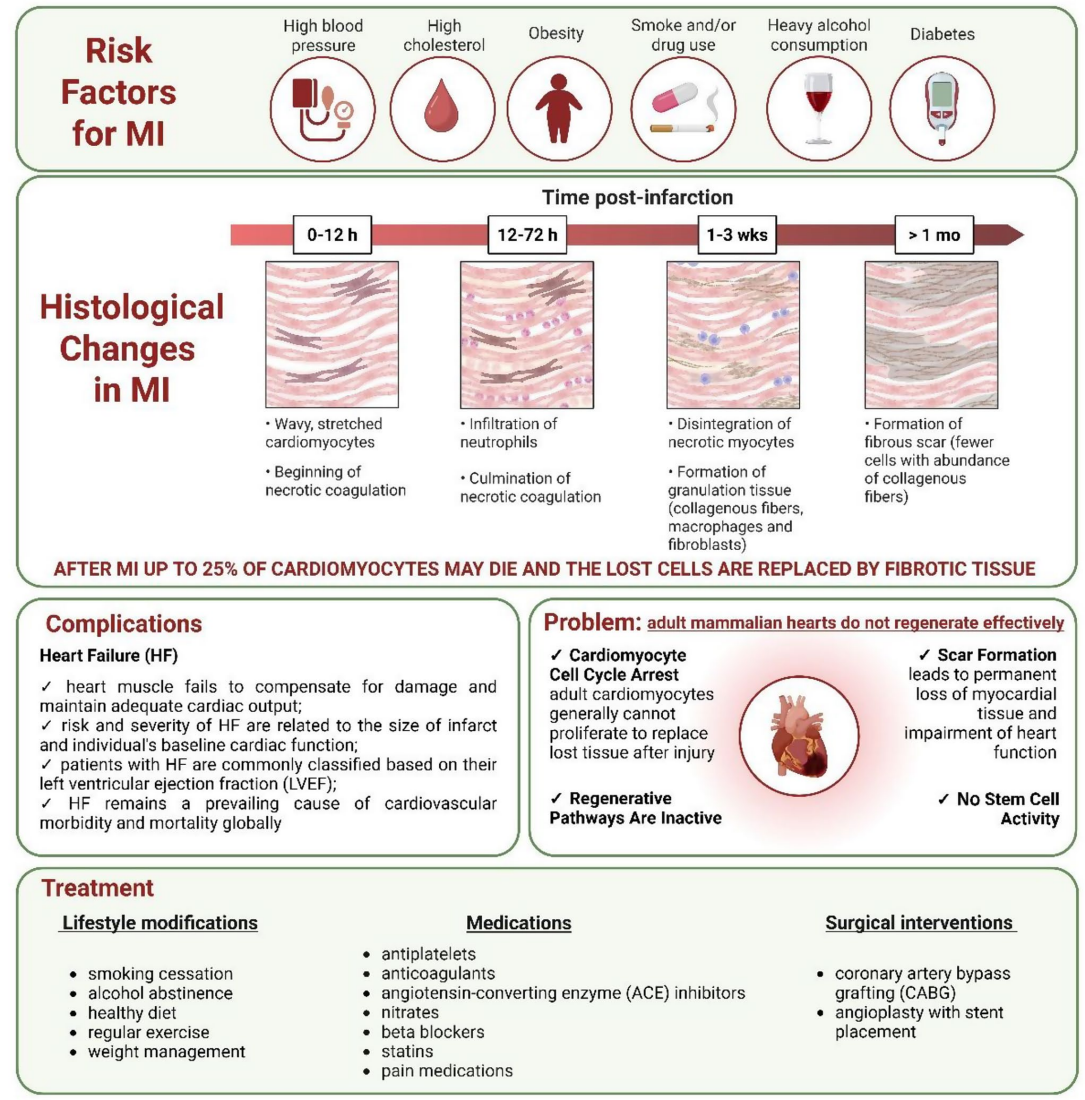
Overview of myocardial infarction. Risk factors, major histological changes, and resulting problems are shown. The limited regenerative capacity of the adult mammalian heart is a consequence of the low proliferative potential of cardiomyocytes, the absence of stem cells, inactive regenerative pathways, and the replacement of damaged myocardium with fibrous tissue. Although several pharmacological and surgical interventions are available, cardiovascular disorders remain the leading cause of death in industrialized and developing countries. HF - heart failure; LVEF - left ventricular ejection fraction; MI - myocardial infarction

Since the 1970s, a better understanding and control of risk factors such as smoking, alcohol consumption, obesity, diabetes, arterial hypertension, and hypercholesterolemia (Fig. 1), combined with new therapeutic procedures, have led to significant progress in the treatment of cardiovascular diseases (CVDs). These advances have notably improved outcomes in conditions such as myocardial infarction (MI), contributing to a gradual reduction in mortality. However, death rates of some disorders, such as heart failure (HF) and sudden cardiac death, remain stable. Generally, CVDs continue to be a major challenge in medical treatment, and are still the leading cause of death in industrialized and developing countries [1].

Following MI, prominent histological changes occur within the cardiac tissue (Fig. 1). Adverse cardiac insults include extensive loss of cardiomyocytes, an intense inflammatory response, degradation of the extracellular matrix (ECM), and subsequent tissue fibrosis. All of these processes contribute to the functional impairment of the heart, ultimately leading to diminished contractile function and the progression of HF [2].

During MI, up to 25% of cardiomyocytes may die, and the lost cells are replaced by fibrous tissue. This is due to the minimal ability of human adult cardiomyocytes to proliferate. This contrasts with the significant cell division that occurs during embryonic development to form a functional heart. Importantly, the heart lacks stem cells capable of generating new cardiomyocytes (Fig. 1). Therefore, the limited regenerative potential of the human heart leads to the impairment of heart functions in patients suffering from MI, followed by HF (see Sadek and Olson [3], Secco and Giacca [4], and Martyniak et al. [5] for reviews).

Currently, despite significant advancements in understanding the risk factors for MI and improving pharmacological treatments, along with lifestyle modifications, and, in some cases, surgical interventions (Fig. 1), the search for therapies that can stimulate the proliferation of endogenous cardiomyocytes remains a critical unmet goal in clinical medicine for HF patients (see Narkhede et al. [6] for review).

This review aims to highlight key discoveries with translational potential that regulate cardiomyocyte proliferation and to discuss how targeted modulation of specific signaling pathways (e.g., Hippo-YAP), cell cycle regulators, epigenetic mechanisms, and extracellular components may support heart regeneration. It is based on a narrative synthesis of current evidence, published up to October 2025, regarding cardiomyocyte proliferation and cardiac regeneration. To ensure a comprehensive and up-to-date overview of the field, a structured literature search was performed conducted across multiple electronic databases, including PubMed, Scopus, and Web of Science, and ClinicalTrials.gov, using combinations of keywords such as cardiac regeneration, cardiomyocyte proliferation, heart repair, cell cycle re-entry, cardiomyocyte renewal, cell-free therapy, microRNA, Hippo/YAP/TAZ, direct cardiac reprogramming, transcription factors, vagal nerve stimulation, and extracellular matrix. A structural search strategy was employed, using a combination of free-text keywords to capture a broad and relevant range of studies. The primary search terms included combinations of keywords such as cardiac regeneration, cardiomyocyte proliferation, heart repair, cell cycle re-entry, cardiomyocyte renewal, cell-free therapy, microRNA, Hippo/YAP/TAZ, direct cardiac reprogramming, transcription factors, vagal nerve stimulation, and extracellular matrix. Only peer-reviewed articles published in English were included. Both experimental and clinical studies were considered, with emphasis on mechanistic insights, translational potential, and interventions relevant to heart regeneration. Review articles were included only when they offered unique perspectives essential for contextual understanding. Reference lists of relevant papers and reviews were also screened to identify additional studies not captured by keyword primary searches. No formal systematic review or meta-analysis was conducted.

### How to study cardiac regeneration?

Cardiac regeneration potential is highly dependent on the vertebrate species and may be influenced by the age/stage of development. It can be robust in fish, amphibians, and neonatal mammals but is limited in adult mammals. The choice of model is therefore crucial for understanding the underlying mechanisms of heart repair and developing potential therapeutic strategies to enhance cardiac regeneration in humans, particularly in the context of adult heart disease.

Numerous studies utilizing ventricular resection, cryoinjury, hypoxia-reoxygenation injury, cauterization, and genetic ablation of cardiomyocytes have been conducted in the zebrafish (*Danio rerio*), renowned for its remarkable regenerative capacity. These injury models elicit varying responses, ranging from partial to complete recovery, with cryoinjury and cauterization closely resembling mammalian MI. On the other hand, research in mammals, using similar approaches (e.g., left anterior descending (LAD) coronary artery ligation, resection injury, ischemic MI, and cryoinjury), demonstrated that there is a narrow window of increased regenerative capacity shortly after birth, which declines with age (see Weinberger and Riley [7] for review and references). Studies in other species also indicate that the severity of the injury plays a significant role in determining the outcome of cardiac regeneration. Interestingly, despite this general phenomenon, some organisms, such as the Japanese rice fish (*Oryzias latipes*), also known as medaka [8, 9], exhibit restricted cardiac recovery. At the same time, adult spiny mice of the genus *Acomys* [10, 11] are characterized by robust cardiac recovery compared to their closely related species. Nevertheless, it is generally accepted that mammals are characterized by a less regenerative potential, largely due to the increased complexity of heart function and higher physiological demand, compared to lower animals.

To investigate the role of specific factors or pathways in the regeneration process, knockout animals or, conversely, overexpression approaches employing lentiviral and adeno-associated vectors for gene delivery were utilized as described below.

### Potential strategies to overcome problems with heart regeneration

In lower vertebrates and neonatal mammals, new cardiomyocytes are generated from pre-existing ones (see Mehdipour et al. [12] for review and references). As a result, current research is focused on understanding the mechanisms that promote the proliferation of adult cardiomyocytes, as well as the molecular pathways that regulate the post-mitotic state of these cells or trigger their division.

### Forcing cardiomyocytes into cell division

The regenerative capacity of the myocardium relies on both responsive cardiomyocytes and pro-mitotic signals, such as heart injury. Bergmann et al. [13] underlined that endothelial cells, mesenchymal cells, and cardiomyocytes are continuously exchanged in the human heart throughout life, though the rates and dynamics of this process vary. While cardiac endothelial cells have the highest turnover rate and the whole population can be renewed approximately every 6 years in adulthood, most postnatal cardiomyocyte turnover is limited to the first decade of life [13].

Many studies have shown that the cardiomyocytes of neonatal mammals retain some proliferative capacity; however, this ability is relatively modest compared to the robust cell division observed during embryonic development. However, injury can stimulate an increase in their proliferative capacity [14, 15]. In adult mammals, tissue damage also triggers mitogenic signals, typically leading to increased polyploidization and multinucleation rather than true cell division [16]. Mitogenic factors that effectively induce significant cardiomyocyte proliferation in lower vertebrates [17] and neonatal mammals [18] are largely ineffective in adult cardiomyocytes. The main obstacle to cardiac regeneration in adult mammals appears to be the cell cycle arrest of cardiomyocytes. Although the myocardium continues to grow during postnatal development and in response to stress, this growth shifts from hyperplastic (cell proliferation) to hypertrophic (cell enlargement), which coincides with the loss of the cardiomyocytes’ ability to divide [19].

A switch in growth modes may help explain why adult mammalian hearts lack regenerative capacity. One theory suggests that if continuously contractile cells were to undergo mitosis, it could compromise the heart’s ability to generate contractile force. While fetal hearts undergo hyperplastic growth, the cell cycle of fetal and early postnatal cardiomyocytes is staggered, ensuring that only a small percentage of cells enter mitosis at any given time [14, 15]. Another hypothesis proposes that the developmental mechanisms enabling neonatal heart regeneration become inefficient or inactive shortly after birth. Upon injury, the adult myocardium attempts to reactivate some of these pathways, often referred to as the “fetal gene program”. However, this reactivation does not reverse cell cycle arrest but instead contributes to the hypertrophic response following injury. The “fetal gene program” remains poorly defined, though several molecular pathways, such as Hippo [20], ERBB2 [21], and Meis1 [22], have been implicated in influencing cardiomyocyte cell cycle regulation. Furthermore, the postnatal environment, characterized by increased oxygen levels and mechanical load, triggers a metabolic shift in cardiomyocytes, which can result in DNA damage, activation of the DNA damage response, and subsequent cell cycle arrest [23]. Recent studies have demonstrated that interventions such as hypoxia preconditioning [24] and mechanical unloading [25–27] can reduce DNA damage and reactivate the cardiomyocyte cell cycle in the adult heart.

**Fig. 2:**
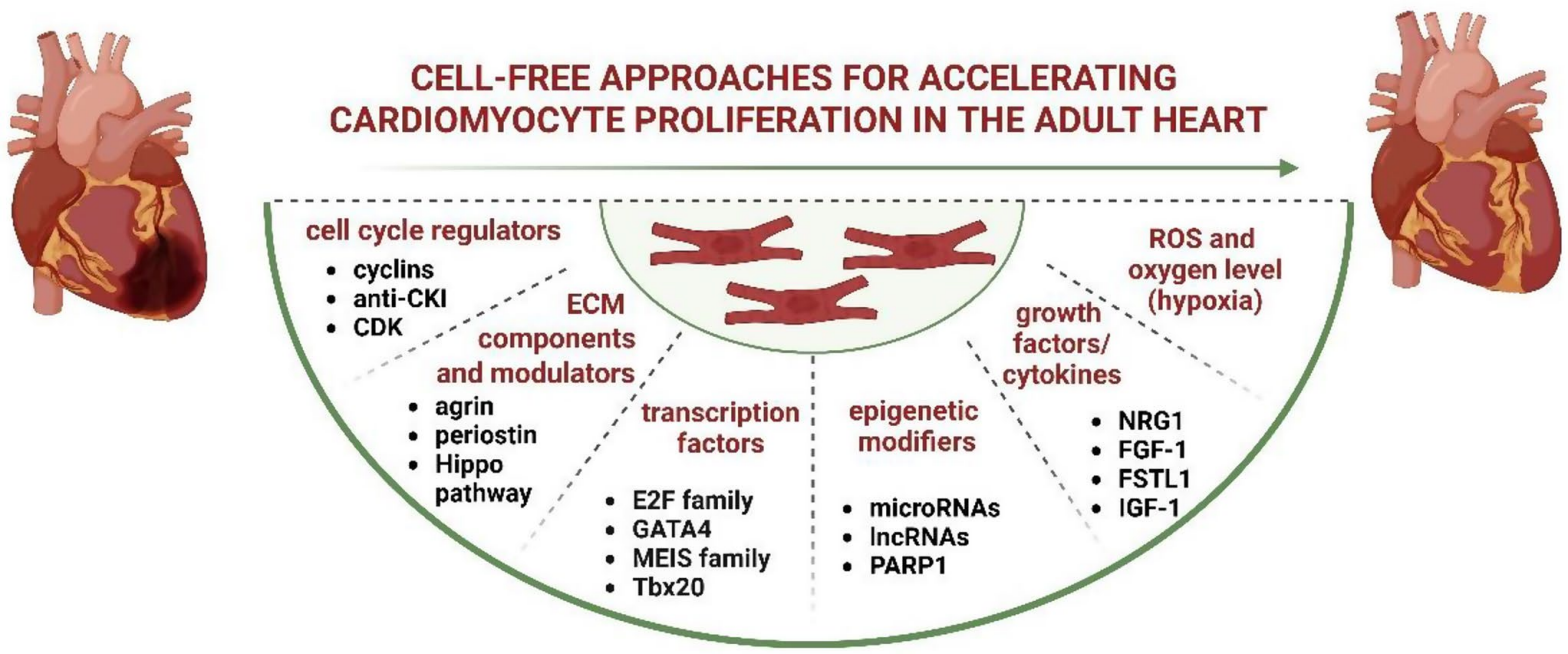
Regulators of cardiomyocyte proliferation. A variety of factors can re-activate cardiomyocyte proliferative and regenerative potential. See the text for further details

The restoration of cardiomyocyte division potential may be influenced by various factors, as illustrated in Fig. 2 and discussed below.

### Cell cycle regulators

**Fig. 3:**
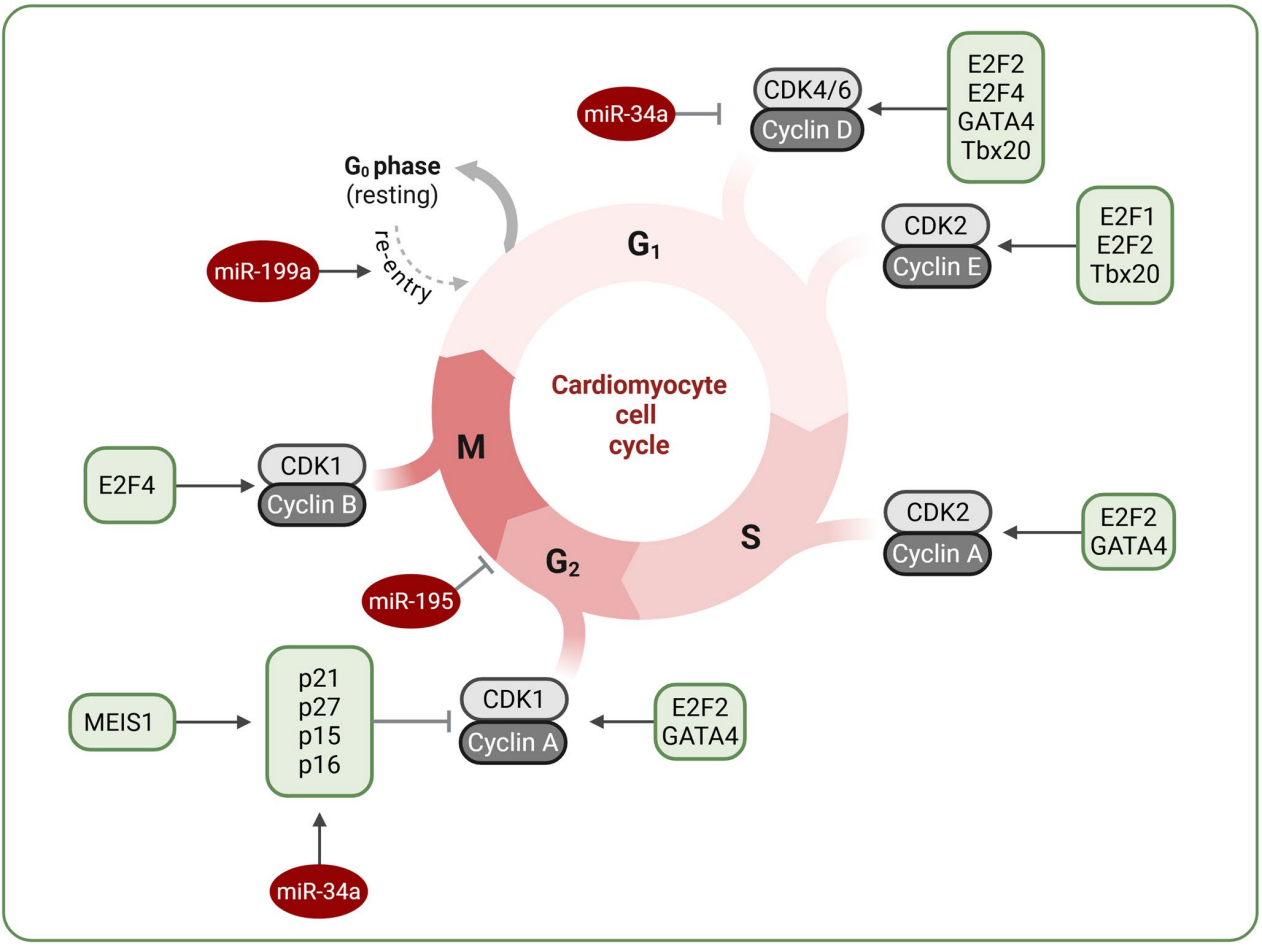
Regulation of cardiomyocyte cell cycle activity. Overexpression of cyclins and cyclin-dependent kinases (CDKs) may offer a promising strategy to stimulate cardiomyocyte proliferation. Additionally, transcription factors such as GATA4 and Tbx20 can promote cell cycle progression. Moreover, MEIS1 can modulate cell cycle activity by increasing the expression of cyclin-dependent kinase inhibitors (CKIs, including p15, p16, p21, and p27). Specific microRNAs, such as miR-34a, miR-195, and miR-199a, also influence cardiomyocyte proliferation

In postnatal cardiomyocytes, the downregulation of cell cycle regulators, such as cyclins and cyclin-dependent kinases (CDKs), coupled with the upregulation of cell cycle inhibitors, including cyclin-dependent kinase inhibitors (CKIs) p15, p16, p21, and p27, triggers the cessation of cardiomyocyte proliferation [28, 29]. Consequently, manipulating the levels of both positive and negative cell cycle regulators presents a promising strategy for cardiac regeneration (Fig. 3).

Pasumarthi et al. [30] demonstrated that constitutively active cyclin D2 overexpression in cardiomyocytes promotes sustained proliferation and myocardial regeneration after injury. Additionally, cyclins D1 and D3 are known to facilitate cell cycle entry [30], while cyclin G1 regulates cardiomyocyte polyploidy and multinucleation, influencing DNA synthesis and inhibiting cytokinesis [31]. Other cyclins, for example, cyclin A2, which is suppressed in postnatal cardiomyocytes [32], have also been studied for their potential to drive cardiomyocyte proliferation. Overexpressing cyclin A2 induced cardiomyocyte proliferation and improved left ventricular (LV) systolic function post-injury in mouse [32] and porcine [33] models. Furthermore, overexpression of cyclin B1, along with cell division cycle 2 kinase (CDC2), stimulated adult rat cardiomyocyte division in vitro [34]. Interestingly, coordinated overexpression of multiple cyclins and CDKs has been shown to induce cardiomyocyte proliferation in the adult mouse heart [35].

### Epigenetic regulators

Over the past few years, epigenetic modulators have emerged as crucial regulators of cardiac regeneration. The number of microRNAs has been explored as potential modifiers of cardiomyocyte proliferation and heart functioning (see Secco and Giacca [4], Braga et al. [36], and Bongiovanni et al. [37] for reviews) (Fig. 3). A high-throughput screening of neonatal rat cardiomyocytes by Giacca’s group [38] revealed 204 miRNAs that significantly increased neonatal cardiomyocyte proliferation. From this group, 40 miRNAs also facilitated EdU incorporation and Ki67 positivity, suggesting that these factors are important regulators of cardiomyocyte mitosis [38]. Among these, the miR-15 family, especially miR-195, which is upregulated in the postnatal heart during cell cycle arrest, inhibits cardiomyocyte proliferation and blocks the G2 phase cell cycle checkpoint [39]. Additionally, down-regulating its expression prolongs the postnatal proliferative window of cardiomyocytes and improves cardiac functions after infarction in young adult mice [15].

Boon et al. [40] hypothesized that the age-dependent decline in cardiac function may result from the disturbed expression of some microRNAs. They found that the expression of miR-34a was approximately twice as high in the hearts of aged (18-20-month-old) C57BL/6 mice compared to young (6-8-week-old) animals. Genetic modifications of miR-34a (silencing with specific antagomir or deletion using miR-34a knockout mice) reduced cardiomyocyte cell death in 18-month-old mice. Additionally, the inhibition of miR-34a expression was beneficial in counteracting MI-induced fibrosis and facilitated recovery of myocardial function. Similarly, Yang et al. [41] also identified miR-34a as a negative regulator of cardiomyocyte proliferation and regeneration in neonatal infarcted hearts and adult mouse hearts. While neonatal cardiomyocytes exhibit a high proliferation capacity, administering a miR-34a mimic to neonatal hearts prevented their proliferation and cardiac recovery after MI. In contrast, treatment of adult animals with anti-miR-34a suppresses its post-MI upregulation and improves myocardial remodeling [41]. These results suggest that modulation of miR-34a may facilitate cardiac repair in adults, indicating its potential clinical significance.

In contrast, miR-199a positively affects cardiomyocyte proliferation, leading to functional heart recovery after injury, as demonstrated in murine [42] and porcine studies [38, 43]. One month after MI and microRNA delivery, treated pigs exhibited increased muscle mass, reduced scar size, and enhanced global and regional contractility. However, despite these short-term benefits, sustained expression of miR-199a led to fatal arrhythmias in the majority of treated animals [43]. These findings underscore a major limitation of pro-regenerative miRNA therapy: while transient induction of cardiomyocyte proliferation can aid in myocardial repair, sustained activation without appropriate temporal and spatial control carries significant arrhythmogenic risk. Therefore, the necessity for tight regulatory control, tissue-specific targeting, and switch-off systems has to be considered in future therapeutic designs. Moreover, the same team recently demonstrated that the timing of AAV6-miR-199a vector administration is critical [44]. In pigs that received the vector 1–2 weeks after MI, no improvement in scar size or function was visible, and, more importantly, many animals died suddenly (40–52 days after vector administration) when the delivery was delayed [44]. This stands in contrast with the beneficial effects seen when the same vector was administered immediately after MI [43]. Additionally, it has to be stressed that the two strands of a miRNA may have opposing functions, as was noted also in the case of miR-199a, where miR-199-3p is pro-proliferative, while miR-199-5p exerts unwanted effects (discussed in [44]). Interestingly, the upregulation of miR-199a in cardiomyocytes is triggered by another microRNA, miR-1825, leading to the repression of the Meis2 transcription factor (a member of the myeloid ecotropic viral integration site family), a negative regulator of cardiomyocyte proliferation [45]. Similarly, the inhibition of Meis1 by miR-548c, miR-509, and miR-23b leads to the proliferation of adult cardiomyocytes [46].

In addition to miRNAs, long noncoding RNA molecules (lncRNAs), which are composed of nucleotides longer than 200, play a critical role in regulating cardiomyocyte proliferation and cardiac repair through interactions with DNA, RNA, or proteins [47]. NR_045363, expressed predominantly in cardiomyocytes, improves cardiac function and stimulates cardiomyocyte proliferation after MI through interaction with miR-216a, and regulation of the JAK2-STAT3 pathway [48]. On the other hand, a cardiac regeneration-related lncRNA (CAREL) was identified as a negative regulator of cardiac regeneration. Its expression in mouse hearts increases from postnatal day 1 (P1) to day 7 (P7) while the regenerative capacity decreases. The cardiac-specific overexpression of CAREL in vivo reduced cardiomyocyte division and proliferation, whereas its silencing enhanced cardiac regeneration and increased heart functions after MI in neonatal and adult mice [49]. Ponnusamy et al. [50] analyzed another lncRNA, cardiomyocyte proliferation regulator (CPR), which exhibits higher expression in the adult heart compared to the fetal stage. When the CPR expression was silenced, cardiomyocyte proliferation in postnatal and adult hearts was increased. Studies performed in CPR-overexpressing and knocked-down cardiomyocytes and CPR-overexpressing and knockout mouse hearts revealed DNA (cytosine-5)-methyltransferase 3 A (DNMT3A) as a direct target of CPR. Interaction of CPR with DNMT3A protein induces CpG methylation of minichromosomal maintenance 3 (Mcm3) promoter and inhibits cardiomyocyte proliferation [50]. Other suppressors of cardiomyocyte proliferation include dachshund homolog 1 (lncDACH1) [51] and natriuretic peptide A antisense RNA 1 (NPPA-AS1) [52], which hamper cardiomyocyte proliferation through binding and interacting with phosphatase 1 catalytic subunit alpha/yes-associated protein 1 (PP1A/YAP1) and splicing factor proline- and glutamine-rich (SFPQ), respectively. Importantly, lncRNAs not only contribute to epigenetic regulation but also play key roles in metabolic reprogramming and miRNA sponging, collectively supporting cardiac regeneration (for references, see [53]). Finally, several circular RNAs (circRNAs), such as circNfix [54], circHipk3 [55], circCDYL [56], and circMdc1 [57], have been identified as key regulators of transcriptional networks controlling cardiomyocyte proliferation and heart regeneration (for more references, see the recent comprehensive reviews [58, 59]).

Additionally, the role of other factors affecting chromatin remodeling has been assessed. Poly(ADP-ribose) polymerase 1 (PARP1), a potent regulator of chromatin dynamics [60], emerged as a modulator of cardiomyocyte proliferation [61]. Knockout of PARP1 negatively impacted scar formation and cardiac functions after apical resection injury, while its overexpression improved heart regeneration. In vitro studies performed on isolated neonatal rat cardiomyocytes revealed significantly increased levels of the cell proliferation markers EdU, Ki67, Aurora B kinase, and phosphohistone H3 (pH3) in cells transfected with adenoviral vectors overexpressing PARP1 compared to control cells. PARP1 activity led to the poly(ADP-ribosyl)ation of heat shock protein 90 alpha family class B member 1 (HSP90AB1), resulting in an increase in binding between HSP90AB1 and cell division cycle 37 (CDC37), which affects cell cycle kinase activity and provides activation of the cardiomyocyte cell cycle [61].

### Transcription factors

Various transcription factors may control cardiomyocyte cell cycle regulation and postnatal cell cycle withdrawal (Fig. 3). Members of the E2F family of transcription factors, known to control the cell cycle, were identified as crucial factors enhancing the proliferation of neonatal cardiomyocytes. The roles of E2F1, E2F2, E2F3, and E2F4 were addressed in various experimental settings, focusing not only on cardiomyocyte proliferation but also on apoptotic pathways. It has been shown that while these factors positively regulate proliferation, they can also simultaneously stimulate cell death in a manner that depends on the specific E2F family member involved [62–64].

GATA4 (GATA binding protein 4), a member of the GATA transcription factor family, is highly expressed in proliferative embryonic and early postnatal cardiomyocytes but is downregulated upon cell cycle arrest. GATA4 overexpression accelerates the proliferation of cardiomyocytes, while its loss inhibits neonatal heart regeneration [65]. Similarly, Tbx20, a member of the Tbx1 subfamily of T-box genes required for embryonic cardiomyocyte proliferation, has been shown to promote heart regeneration in the adult mouse heart, suggesting its critical role in both embryonic and adult cardiomyocyte regeneration [66]. Mechanistically, it inhibits the expression of the antiproliferative genes Btg2, p21, and Meis1 in adult cardiomyocytes, facilitating their proliferation [66]. Notably, the negative impact of members of the MEIS transcription factor family on cardiomyocyte proliferation and cardiac regeneration has been demonstrated. Cardiomyocyte-specific loss of Meis1 enhances cardiomyocyte proliferation in postnatal life and reactivates the cell cycle in the adult heart. This occurs through the transcriptional inhibition of cell cycle inhibitors, including p15, p16, and p21 [22] (Fig. 3). Furthermore, Nguyen et al. [67] demonstrated that deleting Meis and its cofactor, Homeobox B13 (Hoxb13), restored cycle activity in adult cardiomyocytes and improved cardiac functions after MI. Similarly, when Alam et al. [68] inhibited another member of the Meis family, Meis2, in conjunction with the knockdown of Retinoblastoma (Rb1), the proliferation of rat cardiomyocytes isolated from 12-week-old rats and human induced pluripotent stem cell-derived cardiomyocytes (hiPSC-CMs) was enhanced. Additionally, siRNA–mediated silencing of Rb1 and Meis2 in vivo resulted in a reduction in infarct size in rats. When a hydrogel with siRNA-cocktail was intramyocardially injected at multiple sites in the infarcted area, improvement in the cardiac functions and an increase in proliferation indicators, like DNA synthesis marker Ki67, mitotic marker pH3, and cytokinesis marker Aurora B in adult cardiomyocytes were detected [68]. These findings highlight the potential of modulating transcription factors to enhance cardiomyocyte proliferation and cardiac regeneration.

Noteworthy, the overexpression of specific transcription factors, such as GATA4, MEF2C, TBX5, and HAND2, enables the reprogramming of fibroblasts into induced cardiomyocyte-like cells (iCMs) [69–71]. This process recapitulates key aspects of early cardiogenesis, offering a promising strategy for cardiac regeneration. However, the immature phenotype of iCMs, along with challenges in the efficiency and safety of direct reprogramming, may restrict their clinical applicability. Therefore, further optimization of reprogramming cocktails and maturation strategies is needed to fully realize their therapeutic potential (for review, see [72, 73]).

### Growth factors

The number of growth factors can significantly affect cardiac regeneration and cardiomyocyte proliferation, making them possible therapeutic targets for enhancing myocardial regeneration. Neuregulin-1 (NRG1), insulin-like growth factor-1 (IGF-1), fibroblast growth factor-1 (FGF-1), or follistatin-like-1 (FSTL1), a fibroblast-derived growth factor, have been shown to regulate cardiomyocyte proliferation through distinct mechanisms. FGF-1, IGF-1, or FSTL1 primarily act through phosphoinositide-3-kinase/AKT (PI3K/AKT) signaling, while NRG1 activity depends on the interaction with its tyrosine kinase receptors ERBB2 [21, 74] and ERBB4 [75].

In transgenic mice overexpressing human IGF-1 in cardiomyocytes, a significant reduction in cell death in the myocardium was observed one week after coronary ligation, compared to non-transgenic animals [76]. This suggests that IGF-1 plays a protective role in the myocardium following injury by reducing cardiomyocyte apoptosis. Similarly, co-treatment with FGF-1 and SB203580, an inhibitor of p38 mitogen-activated protein kinase (MAPK), accelerated cardiomyocyte proliferation in both neonatal and adult rat models in vitro [77]. In vivo, in the MI model in adult rats, FGF-1 administration facilitated heart regeneration by reducing scar formation and improving cardiac function [78]. Importantly, the effectiveness of both IGF-1 and FGF-1 has been tested in clinical trials. However, the pilot trial (NCT01438086, Table 1) using recombinant human IGF-1 (rhIGF-1, mecasermin) at relatively low doses (1.5 and 15 ng) did not demonstrate statistically significant improvement in left ventricular ejection fraction (LVEF) in ST-elevation myocardial infarction (STEMI) patients [79]. The other clinical trial, NCT00117936, aims to evaluate the effectiveness of multiple intramyocardial injections of the 141-amino-acid form of FGF-1, delivered via a NOGA injection catheter. Although the study mentions low and high doses, the exact dosing parameters are not specified. As of now, no results from this trial have been reported.

FSTL1 is recognized as a pro-regenerative factor that promotes cardiac repair and regeneration. Interestingly, following MI, FSTL1 expression decreases in the epicardium and is instead upregulated in the myocardium. However, myocardium-derived FSTL1 does not enhance regenerative response either under basal conditions or upon transgenic overexpression in cardiomyocytes. On the other hand, administering FSTL1 *via* epicardial collagen nano-fibrillar patches promoted heart regeneration [80]. These differences may be related to the distinct post-translational modifications, such as glycosylation patterns, between epicardial- and myocardial-derived FSTL1, affecting the protein’s structure, receptor binding affinity, and downstream signaling, influencing its biological activity and the context-dependent role in cardiac repair [80].

Interestingly, a disparity also exists in the response of neonatal *versus* adult cardiomyocytes to NRG1, a member of the epidermal growth factor family [18]. In neonatal mice, early treatment with recombinant NRG1 (rNRG1) significantly improved heart function and prevented scarring, whereas delayed treatment was less effective. rNRG1 stimulated proliferation even in binucleated cells, previously considered non-dividing. In pediatric patients under 6 months old with congenital heart defects, rNRG1 also promoted cardiomyocyte proliferation, whereas older patients showed no such effect, highlighting an age-dependent response to therapy [18].

The effect of NRG1 on cardiomyocyte proliferation is mediated through its interaction with tyrosine kinase receptors from the ERBB family [22]. Postnatally, *ErbB2* expression significantly declines, and in the adult heart, its level is relatively low under normal physiological conditions, reflecting the organ’s transition from a proliferative to a more hypertrophic growth mode [81]. Loss of ERBB2 in murine postnatal hearts (CM-specific *Erbb2* knockout) abolishes the pro-mitotic response to NRG1, which can be restored by overexpressing a constitutively active ERBB2 receptor in cardiomyocytes [21]. Similarly, administration of NRG1 has been shown to sustain cardiomyocyte proliferation and enhance cardiac regeneration following MI [75]. Moreover, overexpression of ERBB2 led to the upregulation of the stem/progenitor markers RUNX1 and DAB2 in dedifferentiated cardiomyocytes [82], suggesting that NRG1 signaling induces cardiomyocyte dedifferentiation and reverts them to a more immature, proliferative state. However, some studies did not support the finding that NRG1 can induce cardiomyocyte proliferation in adult mice [83]. Therefore, further investigation is required to fully understand how to modulate the NRG1-ERBB2 pathway and test combinatorial approaches (e.g., NRG1 administration with ERBB2 overexpression) to enhance cardiomyocyte proliferation and promote heart repair following injury. Additionally, the crosstalk between NRG1 and other members of the ERBB family has to be analyzed. In fact, recent research has focused on the NRG1–ERBB4 pathway as a potential intervention for heart failure. Cools et al. [84] screened 10,240 compounds and found eight ERBB4-dimerizing candidates. Detailed testing of the EF‑1 compound revealed that it significantly reduces cardiomyocyte death and attenuates hypertrophy and acts as a cardioprotective agent in vivo in wild-type but not in *Erbb4*-null mice [84]. Based on these and other findings, clinical trials evaluating the efficacy of NRG1 in patients with heart failure have been conducted [85–87]. A Phase I, double-blind, placebo-controlled, single-ascending dose study (NCT01258387) demonstrated improvements in LVEF in patients with HF and LV systolic dysfunction [87]. Phase II trials [85, 86] also showed promising results for NRG1, suggesting its potential as a therapeutic agent in HF therapy (Table 1).

As research progresses, the potential of other growth factors, including bone morphogenetic proteins (BMPs), transforming growth factor-beta (TGFβ), Sonic hedgehog (Shh), vascular endothelial growth factor (VEGF), and ciliary neurotrophic factor (CNTF), is also being explored to determine whether they can stimulate mature cardiomyocytes to re-enter the cell cycle and promote myocardial regeneration (see, for example, Bongiovanni et al. [37] for review).

**Table 1:** Selected clinical trials for heart failure based on treatment with growth factors (based on *clinicaltrials.gov*)

Clinical trial (basic information)	Official Title/ Acronym	Brief Summary/ Description	Intervention/ Treatment	Results/Additional Information/References
NEUREGULIN-1 (NRG1)				
**Number**: NCT01258387 **Status**: Completed **Study Completion**: 2013-03 **Enrollment**: 40	Phase 1, Double-Blind, Placebo-Controlled Study of Single Ascending Doses of GGF2 in Patients With Left Ventricular Dysfunction and Symptomatic Heart Failure	Study to determine the safety, tolerability, pharmacokinetics, and immunogenicity of single intravenous administrations of GGF2 (Glial growth factor 2/ Neuregulin 1β3) in patients with LVEF between 10% and 40% and NYHA functional class II to III HF.	Single intravenous infusions of cimaglermin or placebo were performed in 7 ascending dose cohorts (0.007 mg/kg to 1.512 mg/kg), and 40 patients were then evaluated for adverse events (AEs) for 24 weeks after infusion with study visits at 1, 2, 4, and 12 weeks.	• NRG1 was tolerated except for transient nausea and headache occurring at the higher doses. • Transiently elevated liver transaminases and bilirubin were observed in one patient at the highest planned dose. • A sustained improvement in LVEF over 90 days at the higher doses compared to lower doses or placebo was observed [87].
**Number**: NCT01251406 **Status**: Completed **Study Completion**: 2014-03 **Enrollment**: 67	A Randomized, Parallel, Placebo-controlled, Double-blind Phase IIa Study of Efficacy and Safety of Recombinant Human Neuregulin-1 (Neucardin) in Subjects With Stable Chronic Heart Failure	Randomized, parallel, placebo-controlled, double-blind, multi-center study to assess the safety and efficacy of rhNRG-1, also known as Neucardin, as a treatment for patients with stable chronic heart failure, with a NYHA classification of II or III.	Patients were hospitalized for 10 days and infused subcutaneously with rhNRG-1 at two doses or a placebo.	No published results are available.
**Number**: NCT05949801 **Status**: Recruiting **Study Completion**: 2024-08 **Enrollment (estimated)**: 198	A Multicenter, Randomized, Double-Blind, Placebo-Controlled Phase III Clinical Trial Based on Standard Treatment to Evaluate the Effect of Injectable Neucardin on Cardiac Function and Reverse Ventricular Remodeling in Patients With Chronic Systolic Heart Failure	A multicenter, randomized, double-blind, placebo-controlled Phase III clinical trial to evaluate the effect of rhNRG-1 for injection on cardiac function in patients with New York Heart Association class II-III chronic systolic heart failure, and to confirm its efficacy and safety.	This trial, conducted simultaneously in multiple clinical study sites nationwide, includes 198 subjects, 99 subjects in the treatment group (10 days IV infusion of 0.6 µg/kg/day Neucardin + standard basic therapeutic) and 99 subjects in the placebo group.	No published results are available.
**Number**: ChiCTR-TRC-00000414 **Enrollment**: 44 *The study was registered with the Chinese Clinical Trial Registry* *chictr.org.cn*	A Randomized, Double-Blind, Multi-Center, Placebo Parallel controlled, Standard Therapy Based Phase II Clinical Trial to Evaluate the Efficacy and Safety of Recombinant Human Neuregulin-1 for Injection in Patients with Chronic Heart Failure	A double-blind, randomized study to assess the safety and efficacy of recombinant human neuregulin-1 (rhNRG-1) in CHF patients with NYHA functional class II or III.	Forty-four CHF patients were treated with a placebo or rhNRG-1 (0.3, 0.6, or 1.2 µg/kg/day) for 10 days, in addition to standard therapies.	• Among 44 patients, AEs (mostly mild gastrointestinal symptoms like nausea, vomiting, dyspepsia, diarrhea) incidence was 54.4%, 63.6%, 66.7%, and 100% in the placebo, 0.3-, 0.6-, and 1.2-µg/kg groups, respectively; no serious AEs occurred. • The 0.6 µg/kg dose group showed progressive improvement in LVEF%, along with a reduction in both EDV and ESV after treatment. • However, changes in LVEF%, ESV, and EDV across all groups were not statistically significant, likely due to the small sample size (*n* = 8–11) [85].
**Number**: ACTRN12607000330448 **Enrollment**: 50 *The study was registered with the Australian New Zealand Clinical Trials Registry*,* anzctr.org.au*	The safety and efficacy of recombinant neuregulin-1 (rhNRG-1) in patients with stable chronic heart failure	The study to analyze the acute and chronic hemodynamic responses to recombinant NRG-1 (beta(2a) isoform) in patients with stable CHF (age, 60 ± 2; NYHA II: III, LVEF < 40%).	Fifteen patients on optimal medical therapy for CHF received a rhNRG-1 infusion daily for 11 days. Initially, all patients were administered a single dose of 1.2 µg/kg, and then they received 0.6, 1.2, or 2.4 µg/kg over 12 h on days 2–11.	• The therapy was well tolerated. Serious AEs occurred in two patients (13%). One developed nausea and stomach cramps requiring early discontinuation, which resolved after stopping treatment. Another experienced transient gastrointestinal symptoms and later unstable angina, both resolving without lasting effects. • rhNRG-1 treatment resulted in beneficial acute and chronic hemodynamic effects in patients with stable CHF on optimal medical therapy (ACE inhibitors and beta blockers) [86].
**INSULIN-LIKE GROWTH FACTOR-1 (IGF-1)**				
**Number**: NCT01438086 **Status**: Completed **Study Completion**: 2016-12 **Enrollment**: 47	A Randomised Trial Evaluating the Safety and Efficacy of a Single Low Dose of Intracoronary Insulin-like Growth Factor-1 Following Percutaneous Coronary Intervention for ST-Elevation Acute Myocardial Infarction **Acronym**: RESUS-AMI	A double-blind, placebo-controlled, multidose study to evaluate the safety of recombinant human IGF-1 (rhIGF-1, mecasermin) in STEMI patients with significantly reduced (≤ 40%) LVEF and receiving optimal medical therapy.	This trial compares the effectiveness of intracoronary bolus administration of two doses of mecasermin (1.5 ng or 15 ng) with a placebo group (intracoronary bolus of 0.9% NaCl).	• No acute glycemic, hypotensive, and tachycardia AEs, and no increase in later arrhythmias or any other major adverse cardiac events were observed in IGF1-treated subjects. One death occurred in the higher-dose group (15 ng) on day 10 post-STEMI, likely due to an unrelated ventricular arrhythmia. • LVEF increased in all groups at 2 months compared to baseline, with no statistically significant effect of treatment assignment. • Some positive effects were seen in the higher dose group, compared to both the placebo and the 1.5 ng IGF-1 groups [79].
**FIBROBLAST GROWTH FACTOR-1 (FGF-1)**				
**Number**: NCT00117936 **Status**: Unknown **Study Completion**: 2023-03 **Enrollment** (estimated): 150	Human Recombinant Fibroblast Growth Factor-1 (FGF-1), for the Treatment of Subjects With Severe Coronary Heart Disease, a Placebo Controlled, Double-blind, Dose-varying Study	Treatment with human recombinant fibroblast growth factor-1 (FGF-1, 141 amino acid form) for no-option heart patients with chronic, stable angina with documented coronary artery disease, NYHA functional class III or IV.	Up to ten intramyocardial injections of low or high doses of FGF-1, *via* a NOGA Injection Catheter, compared to the placebo group (up to ten intramyocardial injections of solution not containing FGF-1 *via* a NOGA Injection Catheter).	No published results are available.

Abbreviations: ACE - angiotensin-converting enzyme; AEs - adverse events; CHF - chronic heart failure; EDV - end-diastolic volume; ESV - end-systolic volume; HF - heart failure; LVEF - left ventricular ejection fraction; NYHA - New York Heart Association; STEMI - ST-elevation myocardial infarction

#### Definitions

Enrollment - the number of participants in a clinical study; estimated- the target number of participants that the researchers need for the study.

### Extracellular matrix (ECM) proteins and modulators of ECM

**Fig. 4:**
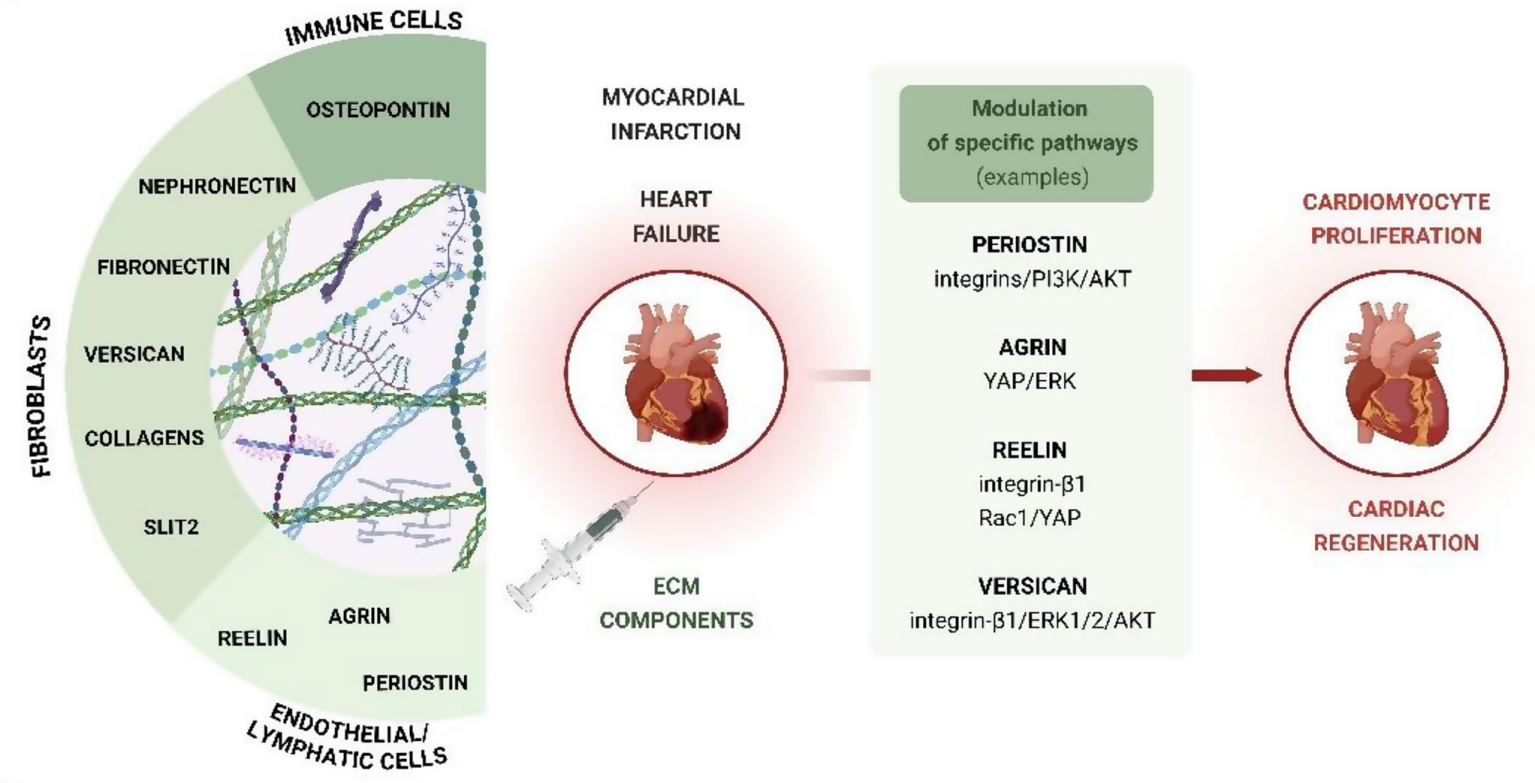
The effect of extracellular matrix (ECM) components on cardiac regeneration and cardiomyocyte proliferation. Fibroblasts, immune cells, and endothelial/lymphatic cells in neonatal hearts produce a variety of ECM proteins, which, through activation of distinct signaling pathways, may facilitate cardiac regeneration in adult animals

Extracellular matrix (ECM) proteins have been widely investigated for their potential to regulate the cell cycle of cardiomyocytes and promote heart regeneration (Fig. 4). The identification of these factors was based on the discovery of prominent differences in heart regeneration in mice between P1 and P7. While the hearts of P1 neonatal mice exhibit a remarkable capacity to regenerate tissue following partial surgical resection of the apex, showing only slight fibrosis or hypertrophy, this regenerative potential is lost by 7 days of age [14]. The simultaneous postnatal decline in ECM protein expression suggests that they may play a critical role in the loss of regenerative potential. Among the various ECM components, agrin [88–91] and periostin [92–96] have emerged as key regulators, owing to their significant influence on cardiomyocyte proliferation and heart regeneration (Fig. 4).

Bassat et al. [88]. showed that agrin, a protein playing a fundamental role in cardiac development [89], is essential for neonatal heart regeneration in mice. Interestingly, direct administration of agrin into the adult myocardium was found to reactivate the cardiomyocyte cell cycle and promote heart regeneration in adult mammals as well. However, the beneficial effects of agrin appear to involve multiple mechanisms and are not solely attributed to stimulating cardiomyocyte proliferation. Mechanistically, agrin may influence cardiomyocyte proliferation through the activation of Yes-associated protein (YAP), which is important for regulating cardiomyocyte division (as discussed below), and through extracellular signal-regulated kinase (ERK)-mediated signaling pathways [88]. Recent studies have also highlighted the role of cardiac fibroblast senescence in neonatal heart regeneration [90, 91], with agrin potentially playing a key role in modulating this process [90].

Another ECM component, periostin, a member of the matricellular protein family, has also been shown to play a role in heart regeneration. Chen et al. [92] discovered that the absence of periostin impairs angiogenesis and promotes excessive fibrotic tissue accumulation in the heart. In neonatal periostin-knockout mice subjected to MI, myocardial fibrosis persisted for up to 3 weeks after injury. In contrast, in wild-type animals, fibrotic tissue was completely replaced by regenerated myocardium during the same period [92]. Moreover, periostin has been shown to stimulate the proliferation of adult rat cardiomyocytes through the activation of αV, β1, β3, and β5 integrins on the cardiomyocyte membrane, as well as the PI3K/AKT pathway, although it does not involve the ERK signaling pathway [93]. However, Lorts et al. [94] reported that modifying periostin expression, either through transgenic overexpression or gene knockout, did not significantly affect myocardial regeneration after infarction when compared to strain-matched control mice. However, periostin has been shown to recruit activated fibroblasts in mice [95] and to increase myocardial fibrosis after experimental MI in swine [96], which limits its potential to promote heart regeneration.

Moreover, other proteins, including specific collagens (e.g., *Col15a1* [97]), nephronectin (NPNT) [98], reelin (RELN) [99, 100], Slit2 [98], and osteopontin (OPN) [101], are enriched in the neonatal cardiac ECM and can promote cardiomyocyte proliferation. Recently, versican, chondroitin sulfate proteoglycan, was identified as an additional potent stimulator of cardiac regeneration, further expanding this group of regenerative ECM factors [102]. Cardiac fibroblast-derived versican was found to be significantly enriched in neonatal mouse hearts 1 day after cardiac apical resection. Functional studies, performed both in vitro (using primary neonatal mouse cardiomyocytes and hiPSC-CMs) and in vivo (in a murine model of conditional knockout of versican in cardiac fibroblasts), demonstrated its key role in promoting cardiomyocyte proliferation and heart regeneration *via* integrin β1 and downstream signaling molecules, including ERK1/2 and AKT [102] (Fig. 4).

In summary, further investigation into how ECM components influence cardiomyocyte proliferation and tissue repair could provide novel therapeutic approaches that lead to enhanced cardiac recovery following injury. Manipulating the cardiac ECM, through local delivery of signaling molecules, the use of biomaterials, or the application of patches, holds significant potential as a strategy for promoting heart regeneration. For a more in-depth discussion of this topic, readers are referred to recent comprehensive reviews [103, 104].

### The Hippo pathway

**Fig. 5:**
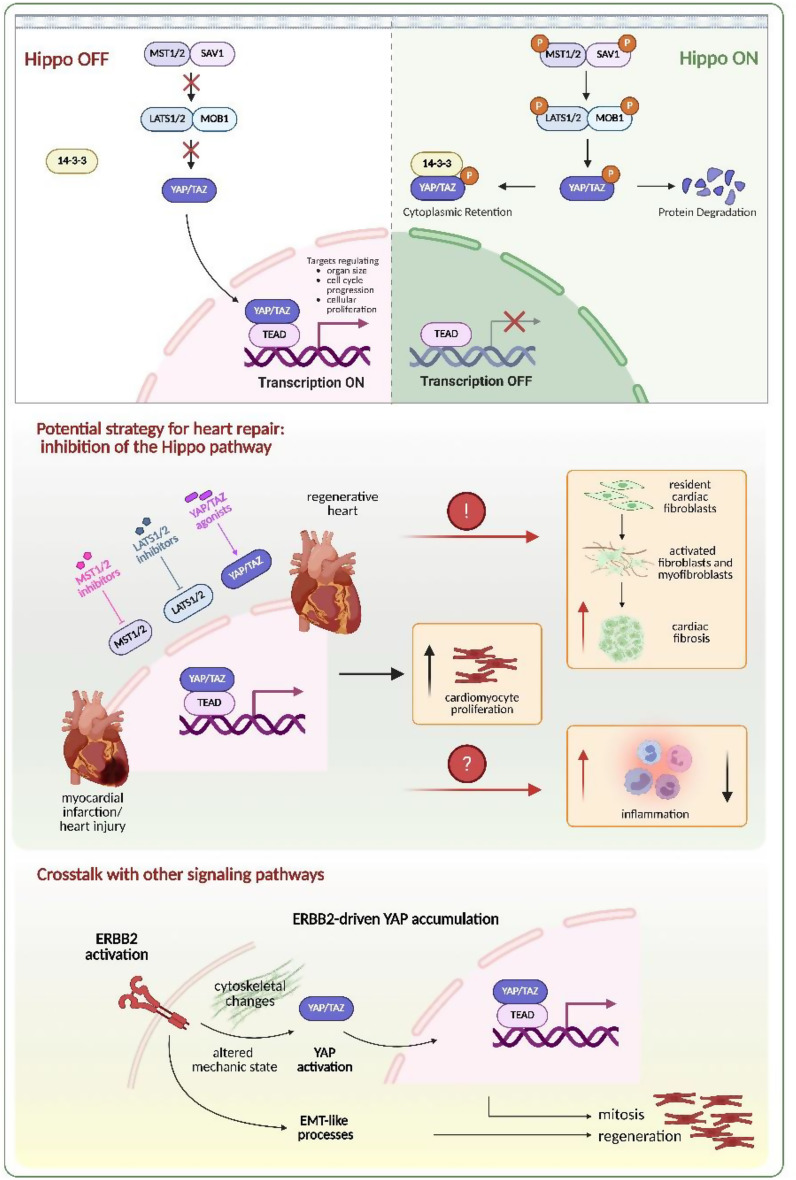
Hippo signaling pathway and its role in cardiac repair. When the Hippo pathway is active, a kinase cascade (involving mammalian STE20-like protein kinase 1/2 (MST1/2) and large tumor suppressor homologue 1 (LATS1/2)) phosphorylates Yes-associated protein (YAP) and transcriptional co-activator with PDZ-binding motif (TAZ), preventing their nuclear localization and transcriptional activity. Conversely, when the pathway is inactive, YAP/TAZ proteins translocate to the nucleus and bind to TEAD transcription factors, promoting the expression of genes involved in cell growth and survival (upper panel). Inhibition of the Hippo pathway through MST1/2 or LATS1/2 inhibitors, or YAP agonists, may enhance cardiomyocyte proliferation. However, it was demonstrated that YAP activation can lead to cardiac fibrosis and has divergent roles in inflammation (middle panel). Crosstalk with other pathways, including ERBB signaling, may also affect the outcome of the YAP on cardiac regeneration (lower panel)

The Hippo pathway is a highly conserved signaling cascade that regulates various physiological processes, including cell proliferation, differentiation, and organ growth (Fig. 5). It plays a critical role in tissue regeneration by mediating cellular responses to mechanical cues, such as ECM stiffness, and regulates the balance between ECM production and degradation [105, 106].

Key downstream effectors of the Hippo pathway, the transcriptional co-activators YAP (Yes-associated protein) and TAZ (transcriptional co-activator with PDZ-binding motif), are involved in regulating the mechanical properties of cells and modulating matrix stiffness and mechano-transduction, both of which significantly influence cell fate [107]. When the Hippo pathway is “on”, the activity of YAP/TAZ is inhibited through large tumor suppressor kinases 1 and 2 (LATS1/2)-mediated phosphorylation and their retention in the cytoplasm. Additionally, 14-3-3 proteins bind to and sequester YAP/TAZ to the cytoplasm, inhibiting their function. On the other hand, when LATS complexes are inactive, unphosphorylated YAP/TAZ translocate to the nucleus. Once in the nucleus, YAP interacts with TEAD transcription factors, which are the terminal effectors of the Hippo signaling pathway. This interaction promotes the expression of genes that drive cell cycle progression and support cellular proliferation, making YAP a key regulator of tissue growth and regeneration [108].

The role of the Hippo pathway in heart regeneration was first highlighted in a 2011 study by Heallen et al. [20], which demonstrated its involvement in regulating cardiomyocyte proliferation during embryonic development and the early postnatal period. Subsequent research has further explored how dysregulation of the Hippo signaling pathway, such as the deletion of scaffold protein Sav1 (also known as Salv1; Salvador homolog 1) or the downstream effectors Lats1 and Lats2, as well as the overexpression of YAP in adult cardiomyocytes, can stimulate cardiomyocyte proliferation and improve heart regeneration following MI injury [109–111]. In particular, Xin et al. [112, 113] demonstrated that mice overexpressing a constitutively active, nuclear form of YAP, generated by substitution of Ser112 with Ala (S112A) and specifically expressed in cardiomyocytes under the control of the αMHC promoter, exhibited a significant hyperplastic response, marked by increased cardiomyocyte proliferation, thickening of the myocardium, and myocardial regeneration after injury. These and other studies, summarized for example in a comprehensive review [114], underscore the importance of the Hippo pathway in controlling cardiomyocyte cell cycle regulation and promoting heart regeneration. Modulating this pathway may offer a promising therapeutic approach for enhancing cardiac repair after injury.

However, Hippo signaling, especially its downstream effector YAP/TAZ, can also regulate the behavior of cardiac fibroblasts and the development of heart fibrosis [115]. Upon injury, cardiac fibroblasts are the predominant non-myocyte cell type that are responsible for synthesizing, degrading, and remodeling the ECM components. During tissue repair, YAP activation, triggered by the loss of LATS1/2, promotes the activation and differentiation of resident fibroblasts into myofibroblasts, thereby contributing to fibrosis and inflammation. Mia et al. [116] demonstrated that YAP/TAZ are markedly upregulated in resident cardiac fibroblasts after MI and are essential mediators of TGF-β–induced pro-fibrotic responses and myofibroblast differentiation. Moreover, YAP/TAZ factors regulate the promoter activity of pro-fibrotic cytokine interleukin-33 (IL-33) and contribute to its secretion by cardiac fibroblasts, thereby promoting myocardial fibrosis [116]. However, Ramjee et al. [117] observed an opposite outcome of the loss of YAP/TAZ in the epicardium, which led to severe pericardial inflammation and myocardial fibrosis after MI, ultimately progressing to cardiomyopathy and mortality. These observations indicate cell-type-specific functions of YAP/TAZ in the processes of cardiac remodeling and repair after MI.

**Fig. 6:**
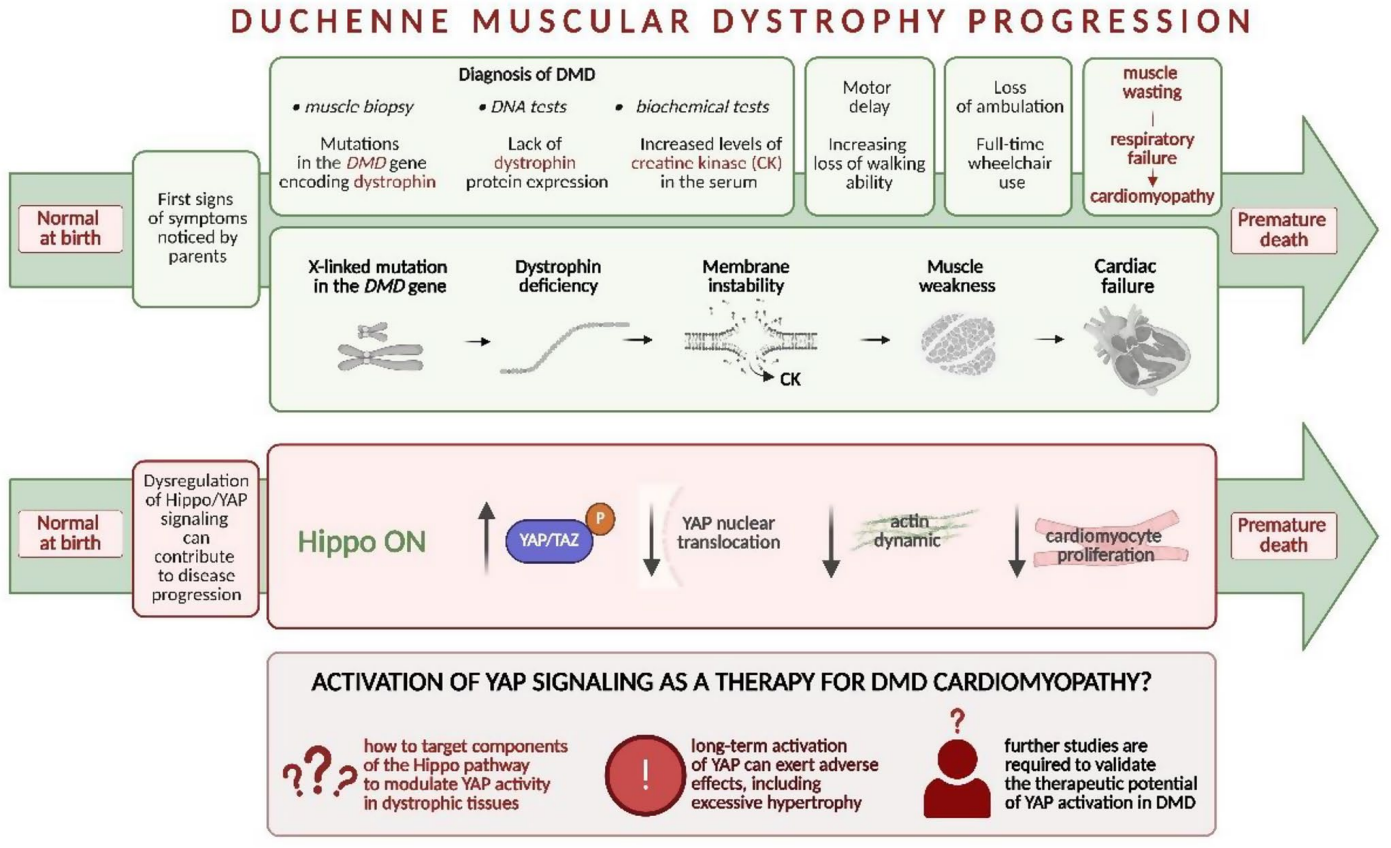
Progression of Duchenne muscular dystrophy and the role of Hippo/YAP signaling. DMD is typically diagnosed in early childhood based on elevated plasma creatine kinase (CK) levels, absence of dystrophin in muscle biopsy samples, and confirmatory genetic testing for specific mutations in the *DMD* gene. The consequence of dystrophin absence and the direct cause of mortality is heart-related perturbations, mostly cardiomyopathy. As a decrease in YAP nuclear translocation can affect the proliferation of cardiomyocytes, its activation could be considered a potential therapy for DMD-related cardiac problems

Additionally, it should be taken into consideration that Hippo signaling may act in parallel with other key regulatory mechanisms in cardiac regeneration. Accordingly, Aharonov et al. [118] investigated the possible crosstalk of the Hippo-YAP pathway and the ERBB2-NRG1 axis. This study revealed that YAP functions as a central downstream effector of ERBB2 signaling, mediating the regenerative and proliferative responses triggered by ERBB2 activation in cardiomyocytes. Mechanistically, ERBB2 activation led to the nuclear accumulation of YAP, which in turn facilitated the transcriptional activation of target genes associated with cell proliferation, cytoskeletal remodeling, and extracellular matrix dynamics (Fig. 6). These findings suggest that the ERBB2-YAP axis represents a converging point in the regulation of cardiac repair, offering potential therapeutic targets for enhancing myocardial regeneration.

Interestingly, the Hippo/YAP signaling pathway is dysregulated not only in typical cardiovascular diseases. For example, it may play a role in the pathogenesis of Duchenne muscular dystrophy (DMD), a progressive, genetic (X-linked recessive) neuromuscular disorder caused by mutations in the *DMD* gene. DMD patients experience progressive muscle degeneration due to the absence of dystrophin, a structural protein essential for maintaining muscle fiber integrity. The lack of dystrophin impairs motor function from early childhood, ultimately leading to loss of ambulation during adolescence [119]. In cardiomyocytes, the absence of dystrophin leads to membrane instability, contributing to cardiac dysfunction. As a consequence, dilated cardiomyopathy (DCM), often progressing to end-stage HF and associated with arrhythmias, can develop in DMD boys, sometimes as early as 6 years of age, and is the leading cause of premature death [120] (Fig. 6).

Currently, there is no curative treatment for DMD-linked HF, and studies trying to evaluate molecular mechanisms of pathological changes/abnormalities are of utmost importance. Decreased YAP signaling in dystrophic muscles, both in the *mdx* mouse model of DMD and in patient-derived samples, has been reported [121]. This reduction has been attributed, for example, to the cytoplasmic sequestration of YAP or impaired nuclear translocation, ultimately leading to a reduced expression of YAP-regulated genes that promote proliferation and survival [121]. Morikawa et al. [122] further showed that the interaction of the dystrophin–glycoprotein complex (DGC) component dystroglycan 1 (Dag1) with YAP is essential for inhibition of cardiomyocyte proliferation in murine hearts. Moreover, a deficiency in Hippo signaling suppressed cardiomyopathy in a model of pressure overload [122]. Finally, to elucidate the role of YAP specifically in dystrophic cardiomyocytes, Yasutake et al. [123] utilized a hiPSC-CM model. Consistent with observation in dystrophic mice and human muscle samples reported by Vita et al. [121], dystrophic hiPSC-CMs exhibited reduced nuclear translocation of YAP compared with control cells. This altered YAP activity was linked to impaired actin dynamics, which, in turn, led to a decrease in the proliferation of DMD hiPSC-CMs.

Collectively, these findings suggest that diminished YAP activity in the dystrophic heart may contribute to the pathogenesis of DMD cardiomyopathy, whereas its activation could exert beneficial effects. Therefore, therapeutic strategies aimed at maintaining the Hippo pathway in an “OFF” state may represent a promising approach for DMD, particularly for its cardiac complications. However, many questions remain regarding the safety and long-term effects of modulating this pathway (Fig. 6).

### Oxidative stress and hypoxia

The link between cardiomyocyte oxidative state and their inherent regenerative ability has been thoroughly investigated. In 2014, Puente et al. [23] found that exposure of newborn mice to mild hypoxia (15% O_2_) promotes cardiomyocyte mitogenesis and protects against oxidative stress, while the postnatal cell cycle arrest of cardiomyocytes is partly mediated by a metabolic shift from glycolysis to oxidative phosphorylation. This shift leads to an increased production of mitochondrial reactive oxygen species (ROS) and oxidative DNA damage, activating the DNA damage response (DDR), including the upregulation of phosphorylated ataxia-telangiectasia mutated (pATM) protein and downstream cell cycle regulators, such as Wee1 kinase. Conversely, inhibition of DDR and ROS production extends the postnatal proliferative window of cardiomyocytes [23]. These results demonstrate that a hypoxic environment during embryogenesis triggers cell cycle progression. Guimarães-Camboa et al. [124] have identified hypoxia-inducible factor 1-α (HIF-1α) as a crucial regulator of the mid-gestational proliferation of a specific population of fetal cardiomyocytes. Therefore, it might be speculated that the low cardiomyocyte turnover in postnatal life, which decreases exponentially with age and is < 1% per year in adults [13, 125], may result from the normoxic environment and higher oxygen level than in developing hearts. Kimura et al. [126] identified a rare population of hypoxic cardiomyocytes in the adult heart, displaying an enhanced proliferative capacity. Single-cell RNA sequencing analysis suggested that prolyl hydroxylases (PHD), negative regulators of HIF-1α protein stabilization, were significantly downregulated in this cardiomyocyte population [126]. However, it remains unclear whether such cardiomyocytes are truly hypoxic or if HIF-1α is stabilized through non-hypoxic pathways.

The loss of proliferative potential in adult cardiomyocytes is associated with a bioenergetic shift from glucose-driven anaerobic glycolysis to oxygen-dependent oxidative phosphorylation of pyruvate and fatty acids [127]. Interestingly, feeding neonatal mice a fatty acid-deficient diet extended the proliferative window of cardiomyocytes up to 3 weeks postnatally, while under normal conditions, cardiomyocytes switch to oxidative phosphorylation and exit the cell cycle by postnatal day 7 [128]. Similarly, the genetic modification (inactivation) of the carnitine palmitoyltransferase (*Cpt1b*) gene, involved in fatty acid oxidation, resulted in an increased level of alpha-ketoglutarate (αKG), an essential cofactor for histone demethylases. In turn, αKG activated the H3K4 demethylase KDM5, leading to the demethylation of H3K4me3 in genes that control cardiomyocyte maturation. This shifts cardiomyocytes into a less mature state and promotes proliferation [129]. Nakada et al. [24] demonstrated that gradual exposure of mice to severe hypoxia, where oxygen levels were reduced by 1% per day, starting from 20.9% to 7% within two weeks and maintained for the next two weeks at this level, reduced the oxidative metabolic phenotype of adult cardiomyocytes. This regimen decreased oxidative DNA damage and promoted cell cycle progression. Notably, hypoxemia followed by an incremental return to normal atmospheric oxygen, achieved through a 2% daily increase in oxygen over one week before contractile function assessment, counteracted the detrimental effects of MI. This approach reduced myocardial fibrosis, improved LV systolic function, and induced a robust regenerative response with a high rate of cardiomyocyte renewal [24]. These findings imply that hypoxia preconditioning may stimulate damaged cardiomyocytes to proliferate. Indeed, exposure to moderate hypoxia to protect against severe hypoxia/ischemia in tissues is suggested as a nonpharmacological, therapeutic approach in various diseases, including cardiovascular disorders, like MI, arterial hypertension, or chronic coronary artery disease [130]. It was demonstrated that hypoxic preconditioning protected cardiac myocytes from cell death during prolonged periods (18–20 h) of severe hypoxia (< 0.5% O_2_) [131].

The proliferation of cardiomyocytes may be influenced by other cell types present in the heart. As endothelial cells and cardiomyocytes can mutually influence each other, Fan et al. [132] investigated whether regulating hypoxia signaling in endothelial cells could affect cardiomyocyte proliferation. Endothelium-specific depletion of PHD2/3 accelerated cardiomyocyte proliferation in neonatal and adult mice. It also promoted functional recovery after MI, indicating that hypoxia signaling in endothelial cells plays a critical role in regulating the cardiomyocyte cell cycle [132].

**Fig. 7:**
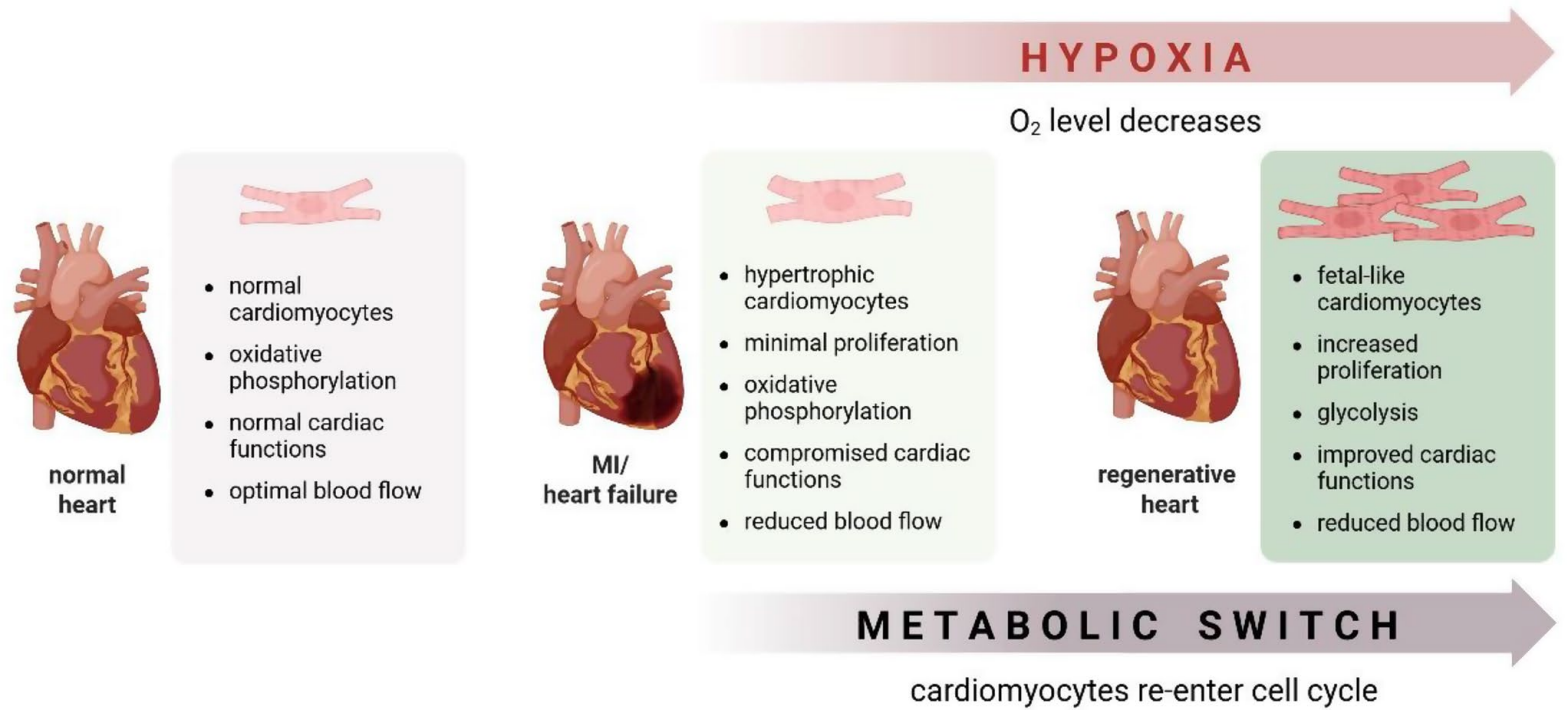
Low oxygen concentration induces heart regeneration. Exposure to progressive systemic hypoxemia can reactivate endogenous regeneration through cardiomyocyte proliferation

These findings collectively suggest that modifying cellular metabolism and oxygen levels can facilitate cardiomyocyte cell cycle progression (Fig. 7). Furthermore, these effects may be influenced by paracrine signals from endothelial cells, underscoring the intricate interplay between various cell types in the heart that can regulate cardiomyocyte regeneration and function.

### Cholinergic innervation as a modulator of cardiomyocyte proliferation

Recent studies suggest that the autonomic nervous system regulates cardiomyocyte proliferation, indicating that neuronal innervation plays a role in controlling heart regeneration in mammals (see Sayers and Riley [133] for a review). Accordingly, manipulating the autonomic nervous system has been shown to regulate neonatal cardiomyocyte proliferation in response to injury [134].

Acetylcholine (Ach), a potent neurotransmitter synthesized by choline acetyltransferase (ChAT), can regulate heart functions through muscarinic ACh receptors (mAChRs) as well as inflammatory pathways and cardiac hemodynamics through nicotinic ACh receptors (nAChRs) (discussed in [135]). Disrupting cholinergic signaling through vagotomy or atropine administration impairs cardiomyocyte proliferation and hinders neonatal heart regeneration, a process mediated by muscarinic M2 receptors. In contrast, cholinergic stimulation has been shown to extend the window for postnatal heart regeneration [134].

A variety of strategies have been investigated to elevate Ach levels. Among them, pharmacological interventions using cholinesterase inhibitors, including donepezil, remain a widely explored option. These agents have been tested in several models of cardiac injury and demonstrated favorable cardioprotective effects (summarized in [135]). Despite these findings, detailed knowledge regarding their potential influence on cardiomyocyte proliferation is lacking. Notably, Kakinuma et al. [136] provided early evidence that cardiomyocytes possess an intrinsic system for intracellular ACh synthesis and demonstrated that donepezil upregulates ChAT promoter activity in these cells. Recent studies on H9c2 rat cardiomyocytes have shown that donepezil protects against a decline in cell viability and necroptosis induced by hydrogen peroxide [137]. Nevertheless, further research is required to clarify the role of cholinesterase inhibitors in modulating cardiomyocyte proliferation and to assess their potential therapeutic implications in cardiac repair.

Cholinergic nerve activation can also be achieved with vagus (vagal) nerve stimulation (VNS), which may be administered using surgically implanted stimulators, external electrical stimulation, or selective targeting of vagal afferent and efferent fibers. This neuromodulatory approach has been proposed as an alternative to pharmacological therapy for HF [138]. The effectiveness of such a strategy has already been tested in vivo and in human trials. While early animal studies suggested the potential benefits of VNS [139], human trials using vagus stimulation devices have shown limited effectiveness, as summarized in Table 2.

**Table 2:** Selected clinical trials for heart failure based on vagus nerve stimulation (VNS) (based on *clinicaltrials.gov*)

Clinical trial (basic information)	Official Title/ Acronym	Brief Summary/ Description	Intervention/ Treatment	Results/Additional Information/References
VAGUS NERVE STIMULATION (VNS)				
**Number**: NCT01385176 **Status**: Active, not recruiting **Study Completion (estimated)**: 2025-06-30 **Enrollment**: 118	NEural Cardiac TherApy foR Heart Failure Study **Acronym**: NECTAR-HF	The NECTAR-HF trial is designed to evaluate the effect of right vagal nerve stimulation on left ventricular remodeling, functional capacity, quality of life, and other measures in heart failure patients with an NYHA Class III, an ejection fraction equal to or less than 35%, over 6 months.	VNS lead was wrapped around the right cervical vagus nerve and then connected to a stimulator permanently implanted in the right pectoral region.	• 87 patients completed the 6-month study, having paired echocardiography exams. • The first six-month randomized phase showed that the safety profile for VNS application was acceptable, with an overall infection rate of 7.4% in the entire cohort [140]. • The 18-month survival rate met the pre-specified safety thresholds and was overall consistent with Seattle Heart Failure Model model predictions, while the 9.4% infection rate aligns with rates reported in the literature [141]. • VNS did not significantly impact the primary and secondary endpoints related to cardiac remodeling and functional capacity in patients with symptomatic heart failure. • Significant improvement in quality-of-life measures was reported [140–142]
**Number**: NCT01303718 **Status**: Terminated **Study Completion**: 2015-12 **Enrollment**: 730	INcrease Of VAgal TonE in CHF - A Randomized Study to Establish the Safety and Efficacy of CardioFit^®^ for the Treatment of Subjects With Heart Failure and Left Ventricular Dysfunction **Acronym**: INOVATE-HF	Prospective, multinational, randomized, open-label, involving 85 centers, event-driven interventional study to evaluate the long-term safety and efficacy of vagus nerve stimulation with the CardioFit^®^ system for treating subjects with chronic HF, NYHA functional class III symptoms, and ejection fraction ≤ 40%.	VNS with the CardioFit^®^ system	• Patients (*n* = 707) were randomized and followed up for a mean of 16 months. • Among 390 implanted patients, 37 had 46 complications within 90 days. Freedom from procedure- and system-related events was 90.6% meeting the first co-primary safety endpoint, while the second endpoint showed no difference in all-cause mortality or complications between groups [143, 144] • VNS did not reduce the rate of death or HF events in chronic HF patients [143, 144]

Abbreviations: HF - heart failure; NYHA - New York Heart Association;

Definition: Enrollment - the number of participants in a clinical study;

For example, results from the NECTAR-HF trial (NEural Cardiac TherApy foR Heart Failure; clinicaltrials.gov: NCT01385176) performed on symptomatic heart failure patients with severe LV systolic dysfunction randomized to receive therapy (VNS) or control (sham stimulation) for a 6-month showed improvements in quality-of-life measures but not in primary and secondary endpoint measures of cardiac remodeling and functional capacity [140–142]. Similarly, the INOVATE-HF trial (Increase of Vagal Tone in Heart Failure; clinicaltrials.gov: NCT01303718) demonstrated some benefit in quality of life and functional parameters, including a 6-minute walking distance test, in patients with heart failure and reduced ejection fraction (HFrEF). However, the LV end-systolic volume index (LVESVI) and the rate of death or HF events in chronic HF patients did not change after VNS [143, 144].

**Fig. 8:**
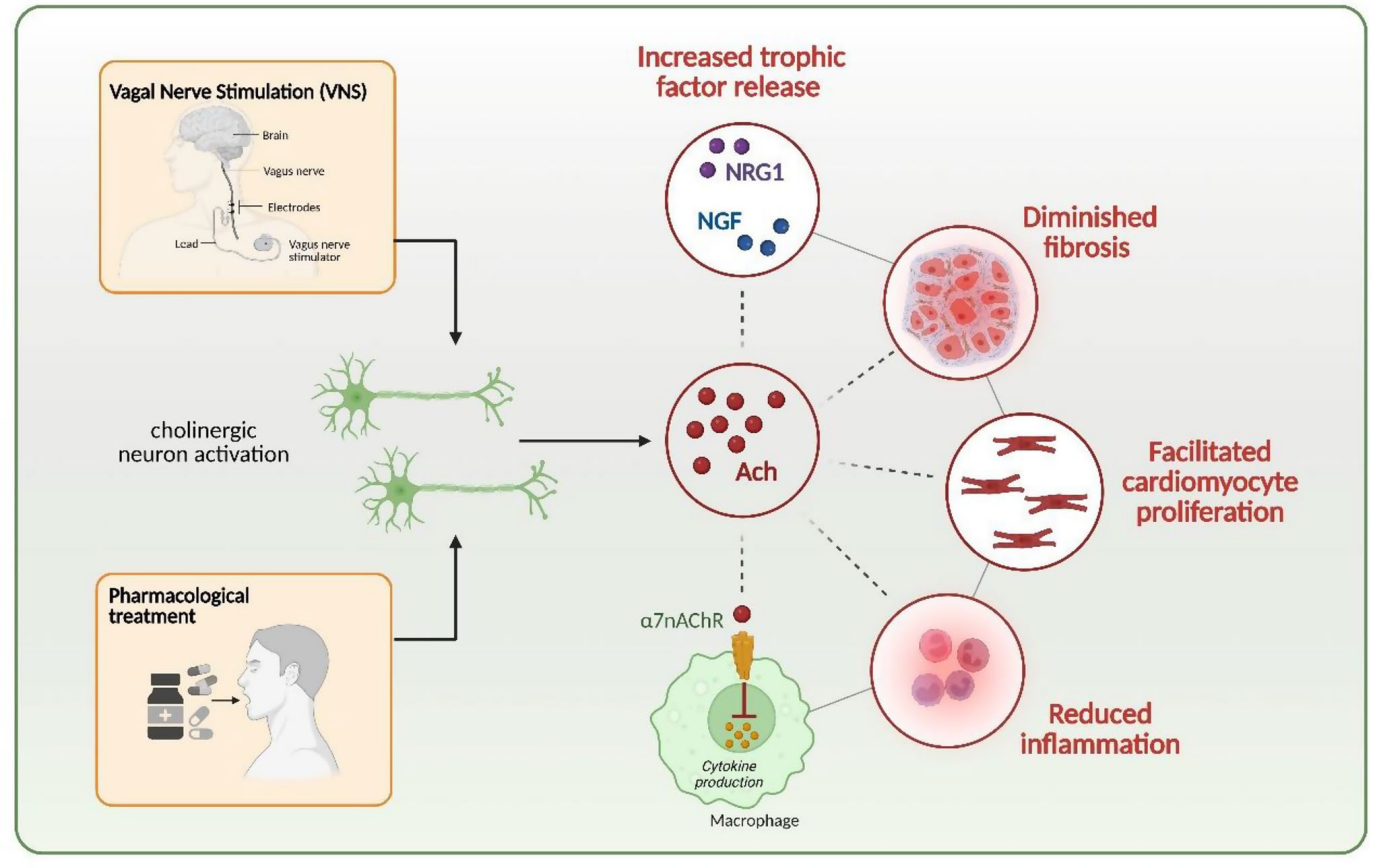
Modulation of cholinergic signaling as a way to increase cardiomyocyte proliferation and heart regeneration. Acetylcholine (Ach), released from cholinergic neurons, is crucial for proper cardiomyocyte proliferation. The activation of cholinergic signaling, for example, by vagus nerve stimulation (VNS) or pharmacological compounds, releases Ach, which in turn exerts beneficial effects on cardiomyocytes and cardiac functions. Moreover, through binding to α7nAchR on macrophages, it can reduce inflammation

Cholinergic signaling can directly influence the cell cycle activity of cardiomyocytes, increasing their ability to proliferate, but may also impact other processes, playing a role during heart recovery. A decline in cholinergic signaling in neonatal mice results in reduced expression of cell cycle genes and lower levels of growth factors NRG1 and NGF. Accordingly, NRG1 or NGF-based therapy partially compensated for the diminished regenerative response caused by denervation. Furthermore, transcriptional profiling of regenerating hearts following vagotomy-induced denervation revealed a notable reduction in the inflammatory response compared to innervated controls [134]. Additionally, it has been proposed that VNS, through activation of α7 nicotinic acetylcholine receptor (α7nAChR), expressed on macrophages, also triggers the cholinergic anti-inflammatory pathway, thereby modulating pro-fibrotic signaling (discussed in [145]). These observations suggest that cholinergic nerve signaling plays a role in regulating cardiomyocyte cell cycle activity by modulating both the expression of key growth factors and the inflammatory environment within the cardiac tissue (Fig. 8).

The sympathetic outflow is essential for neonatal heart regeneration, as evidenced by the regrowth of subepicardial sympathetic nerves during apical regeneration. The role of adrenergic receptors in cardiomyocyte proliferation and regeneration has been addressed in several independent studies. Sakabe et al. [146] demonstrated that metoprolol, a cardio-selective β-blocker for the β1-adrenergic receptor, promoted postnatal cardiomyocyte proliferation and extended the cardiac regeneration window following myocardial infarction, which led to reduced scar formation and improved cardiac function. Similarly, Liu et al. [147]. observed increased cardiomyocyte division in neonatal mice after treatment with the β-blocker propranolol, while Payuma et al. [148] reported that although inhibiting the β-adrenergic receptor alone had only modest effects on cardiomyocyte proliferation and binucleation but the combination of chemical blockers of both α- and β-adrenergic receptors (phenoxybenzamine and propranolol) and modulation of thyroid hormone signaling by propylthiouracil (PTU) delays postnatal cardiomyocyte cell-cycle exit, and the loss of cardiac regenerative potential.

These findings collectively underscore the essential role of cholinergic innervation, as well as the modulation of adrenergic receptor activity, in facilitating effective mammalian heart repair and regeneration.

## Conclusions, limitations, and translational challenges

**Fig. 9:**
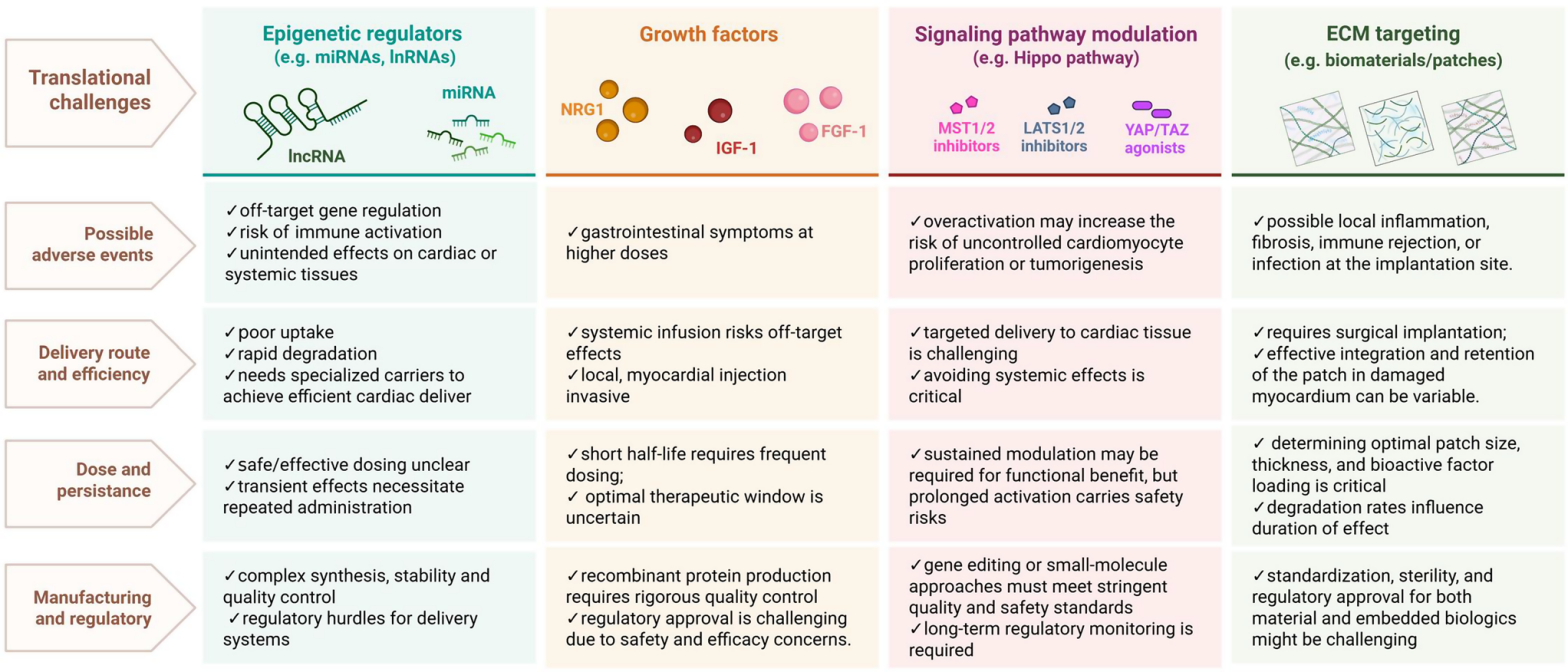
Possible translational challenges of different cell-free strategies for heart regeneration

Cardiomyocyte proliferation and cardiac regeneration occur through a complex interplay of intrinsic factors, including genetic and epigenetic regulators, and extrinsic factors such as growth factors, inflammatory signals, and mechanical stimuli. They can either promote or inhibit cardiomyocyte proliferation depending on the context and type of injury or disease. The balance between these factors determines whether cardiomyocytes enter the cell cycle to regenerate tissue or exit the cycle to maintain heart function. Additionally, signaling pathways influenced by metabolic changes, hypoxia, and interactions between cardiomyocytes and other cell types, such as endothelial cells, further modulate the heart’s regenerative potential. Understanding these regulatory mechanisms, described in this review, offers insights into potential therapeutic strategies for enhancing cardiac repair after injury. However, despite the compelling preclinical evidence supporting the regenerative and cardioprotective effects of cell-free therapies, translation into clinical benefit has been limited. Many preclinical studies are characterized by small sample sizes, short follow-up durations, and inconsistent endpoints, which complicate cross-study comparisons and the assessment of long-term functional outcomes. The use of diverse experimental models, including zebrafish, rodent, and large-animal studies, introduces additional variability due to species-specific differences in cardiac biology, which may also hinder translational applicability to humans. Each of the discussed strategies (miRNAs, growth factors, ECM targeting, Hippo/YAP modulation), although potentially applicable, may encounter significant clinical challenges. These include potential adverse events, challenges in determining optimal dosing and delivery routes, limited therapeutic persistence, and complex regulatory requirements (Fig. 9). These limitations stem from key differences between preclinical and clinical settings, including the frequent use of acute or subacute cardiac injury models that fail to fully recapitulate the complex, chronic pathophysiology of advanced heart failure. Furthermore, patient-specific factors such as comorbidities, disease chronicity, and timing of intervention can further reduce the therapeutic effects observed in animal models. Addressing these translational gaps will be essential for optimizing delivery strategies, identifying responsive patient subgroups, and developing more predictive preclinical models.

Finally, it has to be underlined that this review has its own limitations, such as the possibility of selection bias in study inclusion and the narrative rather than systematic approach to evidence synthesis. Future meta-analyses and rigorously controlled comparative studies will be essential to clarify the therapeutic relevance of the mechanisms discussed and to guide clinical translation.

## Data Availability

No datasets were generated or analysed during the current study.

## Abbreviations

Ach: Acetylcholine
CDKs: Cyclin-dependent kinases
ChAT: Choline acetyltransferase
CKIs: Cyclin-dependent kinase inhibitors
CPR: Cardiomyocyte proliferation regulator
CMs: Cardiomyocytes
CVDs: Cardiovascular diseases
DDR: DNA damage response
DMD: Duchenne muscular dystrophy
ECM: Extracellular matrix
FGF-1: Fibroblast growth factor-1
FSTL1: Follistatin-like-1
GATA4: GATA binding protein 4
HIF-1α: Hypoxia-inducible factor 1-α
HF: Heart failure
hiPSC-CMs: Human induced pluripotent stem cell-derived cardiomyocytes
IGF-1: Insulin-like growth factor-1
LATS1/2: Large tumor suppressor homologue 1/2
mAChRs: Muscarinic acetylcholine receptors
MI: Myocardial infarction
MST1/2: Mammalian STE20-like protein kinase 1/2
nAChRs: Nicotinic acetylcholine receptors
NRG1: Neuregulin-1
PARP1: Poly(ADP-ribose) polymerase 1
pATM: Phosphorylated ataxia-telangiectasia mutated protein
PHD: Prolyl hydroxylases
PI3K: Phosphoinositide-3-kinase
ROS: Reactive oxygen species
TAZ: Transcriptional co-activator with PDZ-binding motif
VNS: Vagus (vagal) nerve stimulation
